# Cationic antimicrobial peptides: alternatives and/or adjuvants to antibiotics active against methicillin-resistant *Staphylococcus aureus* and multidrug-resistant *Pseudomonas aeruginosa*

**DOI:** 10.1186/s12866-019-1416-8

**Published:** 2019-03-08

**Authors:** Regina Geitani, Carole Ayoub Moubareck, Lhousseine Touqui, Dolla Karam Sarkis

**Affiliations:** 1 Microbiology Laboratory, School of Pharmacy, Saint Joseph University, Beirut, Lebanon; 2 College of Natural and Health Sciences, Zayed University, Dubai, United Arab Emirates; 3 Unité de Mucoviscidose et Bronchopathies Chroniques, Institut Pasteur/Faculté de Médecine Cochin, Paris, France

**Keywords:** Methicillin-resistant *Staphylococcus aureus*, Multidrug-resistant *Pseudomonas aeruginosa*, Cationic antimicrobial peptides, Alternative to antibiotics

## Abstract

**Background:**

Methicillin-resistant *Staphylococcus aureus* and multidrug-resistant *Pseudomonas aeruginosa* are becoming difficult to treat with antibiotics whereas Cationic Antimicrobial Peptides (CAMPs) represent promising alternatives. The effects of four CAMPs (LL-37: human cathelicidin, CAMA: cecropin(1–7)-melittin A(2–9) amide, magainin-II and nisin) were investigated against clinical and laboratory *S. aureus* (*n* = 10) and *P. aeruginosa* (*n* = 11) isolates either susceptible or resistant to antibiotics. Minimal Inhibitory Concentrations (MICs), Minimal Bactericidal Concentrations (MBCs), and bacterial survival rates (2 h post-treatment) were determined by microbroth dilution. The antipseudomonal effects of the antibiotics colistin or imipenem combined to LL-37 or CAMA were also studied. The toxicity of CAMPs used alone and in combination with antibiotics was evaluated on two human lung epithelial cell lines by determining the quantity of released cytoplasmic lactate dehydrogenase (LDH). Attempts to induce bacterial resistance to gentamicin, LL-37 or CAMA were also performed.

**Results:**

The results revealed the rapid antibacterial effect of LL-37 and CAMA against both antibiotic susceptible and resistant strains with almost a total reduction in bacterial count 2 h post-treatment. Magainin-II and nisin were less active against tested strains. When antibiotics were combined with LL-37 or CAMA, MICs of colistin decreased up to eight-fold and MICs of imipenem decreased up to four-fold. Cytotoxicity assays revealed non-significant LDH-release suggesting no cell damage in all experiments. Induction of bacterial resistance to LL-37 was transient, tardive and much lower than that to gentamicin and induction of resistance to CAMA was not observed.

**Conclusion:**

This study showed the potent and rapid antibacterial activity of CAMPs on both laboratory and clinical isolates of *S. aureus* and *P. aeruginosa* either susceptible or resistant to antibiotics. Most importantly, CAMPs synergized the efficacy of antibiotics, had non toxic effects on human cells and were associated with transient and low levels of induced resistance.

## Introduction

Infections associated with methicillin-resistant *Staphylococcus aureus* (MRSA) and multidrug-resistant *Pseudomonas aeruginosa* (MDRPA) are becoming difficult to treat due to limited therapeutic options and are requiring alternative antimicrobial strategies [1, 2]. MRSA and MDRPA are part of the world health organization (WHO) list of the families of bacteria posing the greatest threat to human health and for which new drugs are urgently needed [3].

MRSA is constitutively resistant to all ß-lactam antibiotics except cefotetan and ceftaroline due to the *mec*A gene encoding penicillin binding protein PBP2a with a significantly reduced affinity to ß-lactams, rendering these antibiotics ineffective [4]. While several agents including daptomycin, linezolid, tigecycline, and quinupristin/dalfopristin show a certain efficacy against MRSA, vancomycin remains the drug of choice for the treatment of MRSA [5]. Unfortunately, vancomycin-resistant *Staphylococcus aureus* (VRSA) strains have been reported for 15 years and vancomycin-dependent *Staphylococcus aureus* (VDSA) have even been described later [6, 7] proving that surveillance of MRSA-associated hospital and community infections is a serious challenge worldwide [8, 9].

The incidence of hospital acquired infections due to MDRPA, strains defined as non-susceptible to at least one agent in three or more antimicrobial categories [10], has increased and led to high morbidity and mortality in healthcare settings [11, 12]. *P. aeruginosa* infections are often severe, life threatening and difficult to treat because of the limited susceptibility to antimicrobial agents due to the numerous mechanisms of resistance that this organism has accumulated [13]. Multiple studies have demonstrated that resistance to carbapenems, aminoglycosides, and fluoroquinolones, the remaining antibiotics with activity against this Gram-negative bacilli, has critically increased during the past few years [11, 14].

The search for more sophisticated systems to effectively treat multidrug-resistant (MDR) bacteria is essential. Cationic Antimicrobial Peptides (CAMPs) appear to be promising candidates to overcome resistance [15–17]. CAMPs are a large group of low molecular weight natural peptides that play a major role in innate immunity of most living organisms [17, 18]. More than 2400 CAMPs (see Antimicrobial Peptide Database: http://aps.unmc.edu/AP/main.php) have been identified in various species ranging from insects to plants and animals including humans [19]. These agents have a broad spectrum of activity; they exhibit a rapid action against both Gram-positive and Gram-negative bacteria, fungi, viruses, and parasites [17, 20]. Furthermore, CAMPs play a major modulatory role in the innate immune response and support wound healing [21, 22]. Compared to conventional antibiotics, CAMPs cause the death of bacteria quickly by involving many bacterial targets [23]. Mechanisms of action of these peptides vary dramatically; they can either exhibit direct antimicrobial activity or exert a mediator function [24]. CAMPs display a direct activity by disrupting the plasma membrane and/or act on specific intracellular targets to inhibit DNA, RNA or protein synthesis processes, to inactivate essential intracellular enzymes, or to disrupt the plasma membrane formation and cell wall synthesis [25, 26]. One of the major advantages of these peptides lies in their action on both antibiotic susceptible (AS) and MDR bacterial strains [27]. It has been also demonstrated that the efficacy of conventional antibiotics could be further boosted through combination with CAMPs and some studies revealed synergistic relationships between antibiotics and CAMPs [20, 28].

The purpose of this study was to investigate the in vitro antibacterial activities of four CAMPs against clinical and laboratory strains of *S. aureus* and *P. aeruginosa.* We explored the effects of these peptides against both methicillin-susceptible and -resistant *S. aureus* as well as AS and MDRPA strains alone and in combination with antibiotics. The toxicity of antibiotics and CAMPs combinations was evaluated on two human cell lines. The ability of these peptides to induce resistance was also assessed.

This work was, in part, presented orally at the ECCMID 2018 congress (European Congress of Clinical Microbiology and Infectious Diseases) in Madrid, Spain (April, 21–24; presentation number O0253).

## Results

### In vitro antibacterial activity of CAMPs

The in vitro activities of CAMPs LL-37, CAMA, magainin-II and nisin against all *S. aureus* and *P. aeruginosa* are summarized in Table 1. The MIC values obtained were between 2 and > 128 μg/ml. Among the four CAMPs, CAMA had the lowest MICs against both Gram-positive and Gram-negative bacteria, with values ranging between 2 to 8 μg/ml for all tested strains and no major statistical differences between Methicillin-susceptible *S. aureus* (MSSA) and MRSA as well as AS and MDRPA (*P* = 1.000 for *S. aureus* and *P* = 0.545 for *P. aeruginosa*). One clinical MSSA was susceptible to CAMA with an MIC of 2 μg/ml. LL-37 was more efficient on *P. aeruginosa* than *S. aureus*; its MICs varied between 32 and 64 μg/ml for both AS and MDRPA with no significant statistical differences (*P* = 0.242). Besides, magainin-II and nisin displayed MICs higher than 128 μg/ml for all *S. aureus* and *P. aeruginosa* except for two clinical AS *P. aeruginosa* with MICs of magainin-II equal to 128 μg/ml. As shown in Table 1, there were no major differences between MBCs and MICs of the CAMPs tested. MBC values, in the majority of cases, were equal to MIC values. In the cases where they were different, MBC values were only two-fold higher than the MICs.

**Table 1:** In vitro antibacterial activity of cationic antimicrobial peptides against methicillin-resistant and -susceptible *S. aureus,* and antibiotic susceptible and multidrug-resistant *P. aeruginosa*

CAMPs	*S. aureus* (number)	MIC (μg/ml)	MBC (μg/ml)	*P. aeruginosa* (number)	MIC (μg/ml)	MBC (μg/ml)
LL-37	MSSA (5)	> 128	> 128	AS (6)	32-64	32-64
	MRSA (5)	> 128	> 128	MDRPA (5)	32-64	32-64
CAMA	MSSA (5)	2-4	4	AS (6)	4-8	4-8
	MRSA (5)	4	4	MDRPA (5)	4-8	4-8
Magainin-II	MSSA (5)	> 128	> 128	AS (6)	128- > 128	128- > 128
	MRSA (5)	> 128	> 128	MDRPA (5)	> 128	> 128
Nisin	MSSA (5)	> 128	> 128	AS (6)	> 128	> 128
	MRSA (5)	> 128	> 128	MDRPA (5)	> 128	> 128

AS antibiotic susceptible, CAMPs cationic antimicrobial peptides, MDRPA multidrug-resistant *Pseudomonas aeruginosa*, MRSA methicillin-resistant *Staphylococcus aureus*, MSSA methicillin-susceptible *Staphylococcus aureus*

### Colony count 2 h post-treatment

**Fig. 1:**
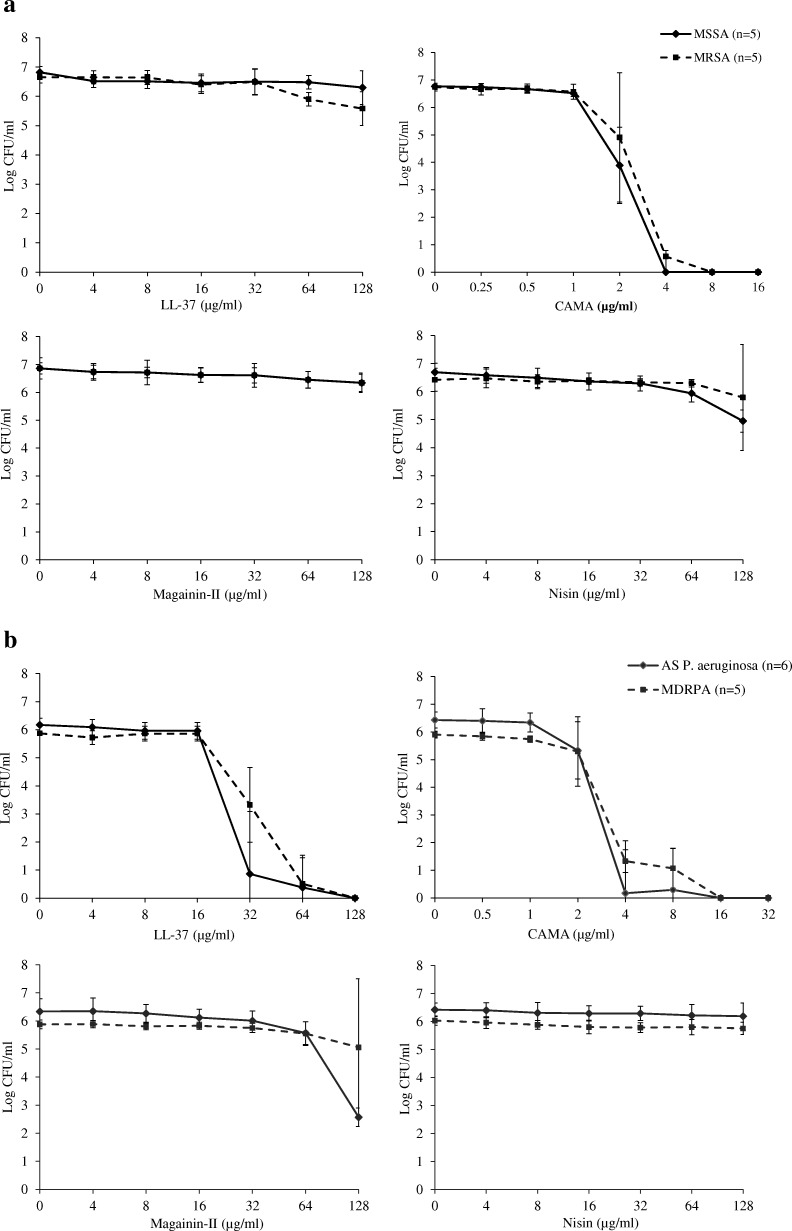
In vitro antibacterial activity of LL-37, CAMA, Magainin-II and Nisin 2 h post-treatment **a**. impact on methicillin-susceptible and -resistant *S. aureus;* **b**. impact on antibiotic susceptible and multidrug-resistant strains of *P. aeruginosa.* The X-axis represents various concentrations of each peptide in μg/ml and the Y-axis represents logarithmic bacterial count. AS: antibiotic susceptible; CFU: colony-forming unit; MDRPA: multidrug-resistant *Pseudomonas aeruginosa*; MRSA: methicillin-resistant *Staphylococcus aureus*; MSSA: methicillin-susceptible *Staphylococcus aureus*

The antibacterial activity of LL-37, CAMA, magainin-II and nisin against tested bacteria was assessed 2 h post-treatment. The killing efficacies of various concentrations of each peptide are shown in Fig. 1a for *S. aureus* and Fig. 1b for *P. aeruginosa*. As shown in Fig. 1a, 2 μg/ml of CAMA resulted in a decrease of approximately 2- to 3-log_10_ of MSSA and MRSA counts respectively with no statistical difference between both groups (*P* = 0.431). Almost a total reduction in bacterial viability was observed at 4 μg/ml of this peptide for both MSSA and MRSA and no statistical difference was obtained between both groups (*P* = 0.141). In contrast, LL-37, magainin-II and nisin had no killing effects on *S. aureus*. As for *P. aeruginosa*, LL-37 and CAMA were found to have significant killing ability at 32 and 4 μg/ml respectively, for both AS and MDRPA strains (Fig. 1b). LL-37 killing efficacy was statistically slightly higher on AS than on MDRPA strains while no statistical differences between both groups were observed for CAMA (*P* = 0.042 for LL-37, *P* = 0.169 for CAMA). Magainin-II and nisin were almost ineffective on both AS and MDRPA strains. The bacterial reduction at 128 μg/ml of magainin on AS strains was due to the susceptibility of two clinical *P. aeruginosa* strains at this tested concentration. Even though magainin-II showed bactericidal activity on two AS *P. aeruginosa* at 128 μg/ml, this efficacy was not statistically significant compared to that on MDRPA strains (*P* = 0.055).

### Activities of CAMPs and antibiotics combinations against *P. aeruginosa*

**Fig. 2:**
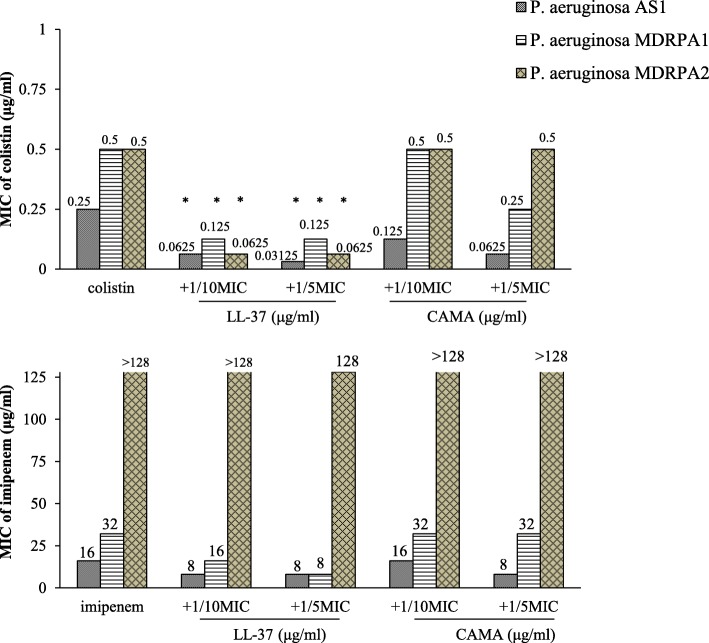
MIC (μg/ml) variations of colistin and imipenem alone or in combination with 1/10 and 1/5 the MICs of LL-37 and CAMA against three clinical isolates of *P. aeruginosa*. AS: antibiotic susceptible, MDRPA: multidrug-resistant *Pseudomonas aeruginosa*. *P < 0.05

The activity of the two most active peptides LL-37 and CAMA were evaluated in combination with colistin and imipenem against three clinical isolates *P. aeruginosa* AS1 susceptible to all tested ß-lactams, *P. aeruginosa* MDRPA1 intermediately resistant to imipenem with a MIC equal to 32 μg/ml and *P. aeruginosa* MDRPA2 strongly resistant to imipenem with a MIC greater than 128 μg/ml. The variations in MICs of tested combinations with peptides are indicated in Fig. 2. When antibiotics were combined with 1/10 × MIC of LL-37, MICs of colistin decreased by four-fold for *P. aeruginosa* AS1 and MDRPA1 and by eight-fold for MDRPA2 while MICs of imipenem decreased by two-fold for AS1 and MDRPA1 and remained greater than 128 μg/ml for MDRPA2. When combined with 1/5 × MIC of LL-37, MIC of colistin for *P. aeruginosa* AS1 decreased by eight-fold and MIC of imipenem decreased by four-fold for MDRPA1. As for strain MDRPA2, the combination of imipenem with 1/5 × MIC of LL-37 decreased the MIC value of this antibiotic to 128 μg/ml. The decrease in MICs due to the combination of colistin with 1/10 and 1/5 × MIC of LL-37 was statistically significant (*P-*values equal to 0.047 and 0.034 respectively). When antibiotics were combined with 1/10 × MIC of CAMA, the MIC of colistin decreased by two-fold for one strain (AS1) out of the three strains while no decrease in the MICs of imipenem was observed. When combined with 1/5 × MIC of CAMA, MICs of colistin decreased by two-fold for *P. aeruginosa* MDRPA1 and four-fold for *P. aeruginosa* AS1 and MIC of imipenem decreased by two-fold for the susceptible strain.

### Synergy studies

To confirm the synergistic activity of antibiotics and CAMPs combinations, checkerboard assays were assessed. The calculated Fractional inhibitory concentration index (FICI) for the tested strains using all the combinations are shown in Table 2. The results indicated synergism between colistin and LL-37 for all the tested strains, either AS or MDRPA, with FICIs < 0.5. For the combination of colistin and CAMA, all three strains showed additive effects with FICIs between 0.5 and 1.5. As for the combination of imipenem and LL-37, only *P. aeruginosa* MDRPA1 showed synergy (FICI = 0.375) while the two remaining strains showed additive results. For the combination of imipenem and CAMA, *P. aeruginosa* AS1 and *P. aeruginosa* MDRPA2 showed additive results (0.5 < FICI ≤ 1.5), while *P. aeruginosa* MDRPA1 showed indifference (FICI = 2).

**Table 2:** MIC (μg/ml) variations of selected antibiotics, CAMPs and antibiotic/CAMP combinations with corresponding FICIs

Strains	MIC			MIC combination		FICI	
	colistin	LL-37	CAMA	col/LL-37	col/CAMA	col+LL-37	col+CAMA
*P. aeruginosa* AS1	0.25	32	4	0.0625/4	0.125/1	0.375	0.75
*P. aeruginosa* MDRPA1	0.5	64	4	0.125/4	0.25/2	0.3125	1
*P. aeruginosa* MDRPA2	0.5	64	8	0.125/8	0.25/2	0.375	0.75
	imipenem	LL-37	CAMA	imp/LL-37	imp/CAMA	imp+LL-37	imp+CAMA
*P. aeruginosa* AS1	16	32	4	8/8	4/2	0.75	0.75
*P. aeruginosa* MDRPA1	32	64	4	8/8	32/4	0.375	2
*P. aeruginosa* MDRPA2	256	64	8	128/16	128/4	0.75	1

Note: FICI was defined as follows: FICI ≤0.5, synergy; 0.5 < FICI ≤1.5, additive; 1.5 < FICI ≤ 2.0, indifference; FICI > 2, antagonism

*AS* antibiotic susceptible, *FICI* fractional inhibitory concentration index, *MDRPA* multidrug-resistant *Pseudomonas aeruginosa*

### Percentage of cytotoxicity

**Fig. 3:**
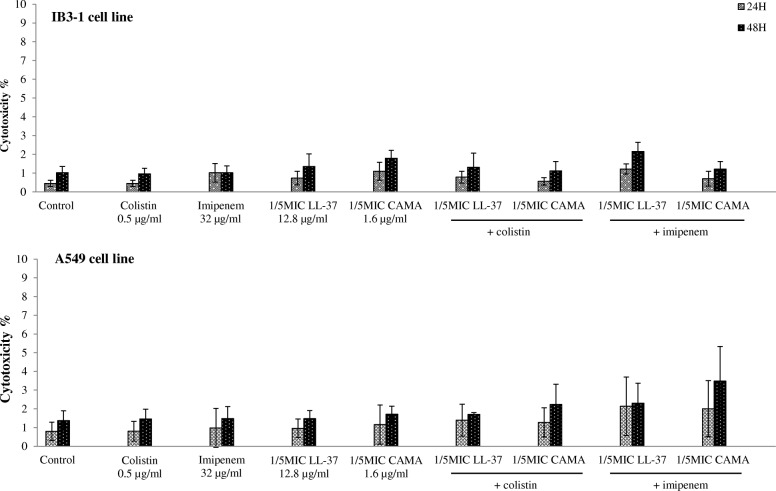
LDH-based cytotoxicity percentages of colistin, imipenem, LL-37, CAMA and antibiotic/CAMP combinations on IB3–1 and A549 cell lines 24 and 48 h post-treatment

CAMPs (LL-37 and CAMA) were screened for their cytotoxicity when given alone and in combination with antibiotics (colistin and imipenem), based on the LDH release from the cytosol of IB3–1 and A549 cell lines 24 and 48 h post-treatment. When tested alone, both CAMPs were not associated with a cytotoxicity effect on both cell lines. Cytotoxicity % were equal to 4.7 and 4.3% for LL-37 (64 μg/ml) and CAMA (16 μg/ml) respectively on IB3–1 at 48 h of treatment. As for A549, cytotoxicity % were equal to 3.4% for both LL-37 and CAMA at 48 h. When combined to antibiotics (Fig. 3), all the obtained percentages were less than 5% and the cytotoxicity % of the tested combinations were not significantly different than those obtained for cells treated with antibiotics or CAMPs alone (*P* > 0.05). A low effect was noticed on A549 cells for imipenem and CAMA combination 48 h post-treatment. Our results showed that the tested concentrations of LL-37 and CAMA, as well as the tested combinations, had minimal cytotoxic effects on both IB3–1 and A549 cell lines 24 and 48 h post-treatment.

### Resistance studies

**Fig. 4:**
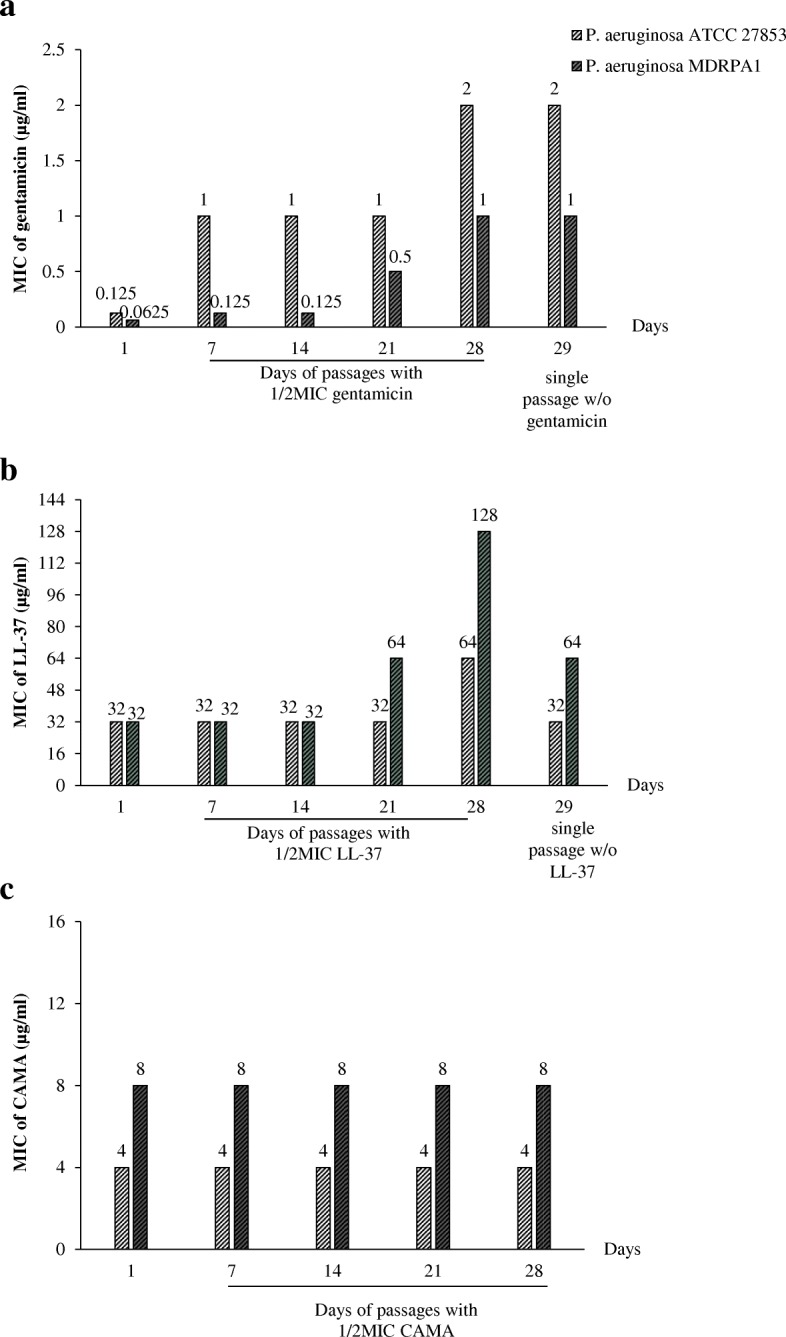
MIC (μg/ml) variations of **a**. Gentamicin, **b**. LL-37 and **c**. CAMA against two *P. aeruginosa* treated daily with half the MIC of these antimicrobial agents. MDRPA: multidrug-resistant *Pseudomonas aeruginosa;* w/o: without

The reference strain *P. aeruginosa* ATCC® 27853™ and the clinical *P. aeruginosa* MDRPA1 were treated daily with gentamicin, LL-37, and CAMA at a concentration equal to half the MIC of each agent. The weekly changes in MICs are shown in Fig. 4a, b and c for gentamicin, LL-37, and CAMA respectively. After 28 passages, the MICs of gentamicin increased by eight-fold for both strains (Fig. 4a), while the MICs of LL-37 increased by two-fold for the reference strain and by four-fold for *P. aeruginosa* MDRPA1 (Fig. 4b). Serial treatments with CAMA had no significant effect on the MICs of this peptide (Fig. 4c). Gentamicin-induced resistance occurred at week 1 for both reference and clinical strains (Fig. 4a). LL-37-induced resistance appeared at week 3 for the clinical strain and at week 4 for the reference strain. LL-37 resistance was transient since a single passage of the resistant strains in the absence of peptide led to a decrease of the MICs by two-fold (Fig. 4b). In contrast, gentamicin-induced resistance in both strains persisted after a single passage without the antibiotic (Fig. 4a).

## Discussion

Antibacterial activities of CAMPs are becoming the focus of numerous studies due to their high potency and rapidity in destroying bacterial cells [17, 19]. We have investigated the in vitro activity of four CAMPs against MSSA and MRSA, and AS and MDRPA strains. The activities of two of these peptides were evaluated in combination with antibiotics. Cytotoxicity of CAMPs when given alone and in combination with antibiotics was analyzed and the ability of two peptides to induce bacterial resistance was also assessed.

CAMA was the only active peptide against laboratory and clinical *S. aureus* strains with MICs equal to 4 μg/ml for both MSSA and MRSA strains. As for *P. aeruginosa,* CAMA and LL-37 were active against the strains. Interestingly, no major differences were noted between MICs and MBCs of these CAMPs against bacteria independently from their antibiotic resistance patterns. This comparative study may be of high importance since it reports that expression of studied antibiotic resistance mechanisms does not alter the efficacy of CAMA and LL-37 against pulmonary pathogens. CAMA and LL-37 may be considered as bactericidal agents since the MBC values were in most of the cases equal to those of the MICs. In agreement with previous findings [29, 30], our results confirm the efficacy of these two peptides in killing laboratory and clinical strains of *S. aureus* and *P. aeruginosa* while conventional antibiotics failed. In contrast, the results concerning magainin-II and nisin showed almost no potency. While the results obtained for magainin-II are in full agreement with previous studies, those for nisin are opposed [20, 30].

MIC and MBC values showed the effects of CAMPs against *S. aureus* and *P. aeruginosa,* but did not provide any evidence on the time required by the peptides to achieve their antimicrobial activity and, therefore, to overcome this issue, enumerations were performed [29, 30]. Our results demonstrated that CAMA and LL-37 tend to show some killing effects at concentrations equivalent to half the MICs and achieve killing quite of all bacteria within 2 h at concentrations equal to MICs (Fig. 1). This reduction in bacterial viability not only confirms the accuracy of the obtained MIC and MBC values but also show the rapid bactericidal effects of these peptides against both laboratory and clinical strains with no major differences between AS and MDR pathogens (*P* > 0.05).

An innovative approach that is gaining interest is the use of combinations of an antimicrobial agent with a CAMP to evaluate their synergistic effects [20, 29]. We investigated the in vitro activity of colistin and imipenem with LL-37 and CAMA separately at 1/10 and 1/5 of the MIC values of each CAMP. The MIC values of these antibiotics decreased up to eight-fold when combined to CAMPs indicating synergism. These results suggest that CAMPs could be used as tools to enhance antibiotic effectiveness against bacteria and decrease their toxicities by lowering the dose required for therapeutic benefit. When added to colistin, a minor amount of LL-37 was able to significantly enhance the antibiotic activity and decrease its MICs by up to eight-fold. This result was observed against both AS or MDRPA strains. The synergistic activity of colistin/LL-37 and colistin/CAMA combinations was further confirmed using checkerboard technique (FICIs < 0.5). Colistin targets Gram-negative bacteria by interfering with the lipopolysaccharide (LPS) of the outer membrane; an essential component of the cell wall [31]. Polymyxins have traditionally been used as last resort to treat serious infections such as those caused by *P. aeruginosa*. Acquired resistance to polymyxins is not common. Only in late 2015, the first transferable plasmid-borne resistance gene to colistin (*mcr-1)* was discovered. More recently, novel colistin resistance genes, *mcr-2*, *mcr-3*, *mcr-4* have been reported [32–34] causing significant concerns due to heavily compromised therapeutic options [35]. The CAMPs tested in this study have a direct action on the bacterial membrane [20]. The overall net negative charge of the bacterial outer membrane makes it an ideal target for these CAMPs. The initial attraction occurs through electrostatic bonds between these cationic peptides and the negatively charged phospholipids in the outer membrane of Gram-negative bacteria. In Gram-positive bacteria, attraction occurs between CAMPs and peptidoglycan components [36]. The CAMPs-induced stress on the lipid bilayer results in destabilization of the bacterial structure, thus allowing the intracellular uptake of antibiotics. In addition to its membrane-directed antimicrobial activities via ion-permeable channels formation, LL-37 binds and neutralizes the LPS with effectiveness comparable to that of polymyxin B; the well-recognized LPS-binder [37]. The synergistic effect of LL-37 with colistin was likely due to the combined effect of these two agents on a shared target resulting in a significant decrease in the MIC values of colistin. The combinations with imipenem were less effective; that could be attributed to the fact that ß-lactams target the PBPs, which are involved in the cross-linking of the bacterial cell wall and are distinct from the target of LL-37. Therefore, adding a minor amount of LL-37 to imipenem was most likely not sufficient to improve significantly the potency of this antibiotic. CAMA is a hybrid peptide that forms ion-permeable channels in model lipid membranes [20]. This peptide had antimicrobial activity against both Gram-positive and Gram-negative bacteria alone but this activity was only marginally enhanced by combination with antibiotics. Synergistic effect of CAMA with antibiotics is mainly due to the greater access of antibiotics to the cytoplasmic membrane resulting from the direct action of CAMA on the outer membrane. Hence, the minor amount of this peptide added to colistin or imipenem did not have a major effect.

Although the use of combinations of antibacterial agents is nowadays of importance, to our knowledge, the possible cytotoxicity of these combinations was not analyzed on human lung epithelial cells. We demonstrated in this study the nontoxic effect of the tested CAMPs alone and in combination with antibiotics on human cell lines 24 and 48 h post-treatment. Our results confirm the non cytotoxic effect of LL-37 at 24 h of treatment as elsewhere reported [38] and at 48 h of treatment. In fact, general toxicity of LL-37 to eukaryotic cells is reported at concentrations > 65 μg/ml [39] while the tested concentration of LL-37 in this study is 64 μg/ml. No data are available to date concerning the cytotoxicity of CAMA on human lung epithelial cells. CAMA is a cecropin-melittin peptide found to display important bactericidal effects against many bacteria. Few publications in this field have reported the limited cytotoxicity effects of cecropin and melittin alone. Our results display, for the first time, the absence of cytotoxicity of this hybrid peptide on two human lung epithelial cells for 48 h.

Besides, strains of *P. aeruginosa* had, respectively low or no potential to develop resistance to LL-37 and CAMA at sub-MICs. In addition, LL-37-induced resistance was reversible by contrast to gentamicin-induced resistance. After daily passages in the presence of sub-inhibitory concentrations, the MICs of LL-37 increased by two- to four-fold after 21 days of passages while resistance to gentamicin occurred at day 7 of treatment resulting in an increase of the MIC values for this antibiotic by eight-fold. Also, no induced resistance was observed for CAMA.

## Conclusions

In conclusion, CAMA exhibited the highest and broadest spectrum of activity against both Gram-positive and Gram-negative bacteria with no significant differences between strains showing various antibiotic resistance phenotypes. LL-37 demonstrated potent antibacterial activity on both laboratory and clinical *P. aeruginosa* strains. The negligible cytotoxic effects of these two CAMPs and their reduced tendency to develop resistance makes them interesting alternative drugs, exhibiting strong and rapid antibacterial activity either alone or in combination with antibiotics. Nevertheless, CAMPs may represent potential future therapeutic solutions for infectious diseases associated to multi-resistant bacteria.

## Materials and methods

### Antimicrobial peptides and antibiotics

CAMPs LL-37, CAMA and magainin-II were obtained from Bachem AG (Bubendorf, Switzerland) and nisin from Sigma-Aldrich (Saint Louis, MO, USA). The antibiotics used were gentamicin (PAYAL, London), colistin sulfate (Sigma-Aldrich), and imipenem (Merck Sharp & Dohme B.V., Haarlem, Netherlands). Antimicrobial powders were dissolved in sterile water at a concentration of 2560 μg/ml then aliquoted and stocked at − 20 °C before use. Diluted solutions were prepared on the day of use. Peptides dilution was made in 0.01 to 0.02% of acetic acid (Sigma-Aldrich) containing 0.2 to 0.4% of bovine serum albumin (Sigma-Aldrich).

### Bacterial strains

A total of 21 bacterial strains were tested and consisted of 10 *S. aureus* and 11 *P. aeruginosa*. Eight clinical isolates of each bacterial species were obtained from human sputum samples sent to Microbiology Laboratories of different Lebanese Hospitals: Hôtel Dieu de France Hospital, Saint George Hospital, Bellevue Medical Center, and Arz Hospital. The following laboratory strains were obtained from the American Type Culture Collection (ATCC): *S. aureus* ATCC® 29213™ and ATCC® 43300™, *P. aeruginosa* ATCC® 27853™, ATCC® 15692™ and ATCC® 53308™.

### Cell lines and culture conditions

Human bronchial epithelial IB3–1 cell line ATCC® CRL-2777™ and human non-small cell lung adenocarcinoma A549 cell line ATCC® CCL-185™ were purchased from the ATCC. IB3–1 cells, derived from a Cystic Fibrosis patient with a ΔF508/W1282X mutant genotype and immortalized with adeno12/SV40, were grown in LHC-8 (Gibco) supplemented with 10% of fetal bovine serum (FBS) (Sigma- Aldrich), 100 units/ml of penicillin and 0.1 mg/ml of streptomycin (Gibco), 1 mM of hepes Buffer (Sigma) and 2 mM of L-Glutamine (Gibco) at 37 °C/5% CO_2_. A549 cells were cultured in Dulbeccos modified Eagles medium (DMEM) with Glutamax (Gibco) supplemented with 10% FBS, 100 units/ml of penicillin and 0.1 mg/ml of streptomycin, at 37 °C/5% CO_2_.

### Characterization of the isolates

Antibiotic susceptibility was assessed by disc diffusion according to the EUCAST (European Committee on Antimicrobial Susceptibility Testing) recommendations [40]. Antibiotic discs and MIC test strips were obtained from BIO-RAD (Marne-la-Coquette, France) and Liofilchem (Roseto degli Abruzzi, Italy) respectively.

The antibiotic susceptibility patterns of the clinical and laboratory *S. aureus* and *P. aeruginosa* are shown in Table 3. Four clinical isolates of *S. aureus* were considered as MRSA due to their resistance to cefoxitin, while the remaining clinical strains were considered as MSSA. The ATCC *S. aureus* ATCC® 29213™ and ATCC® 43300™ were respectively the reference for MSSA and MRSA strains. Certain MRSA strains were resistant to erythromycin, quinolones, fusidic acid and rifampicin. Five clinical *P. aeruginosa* isolates classified as MDRPA were resistant to ß-lactams including third-generation cephalosporins, imipenem and meropenem, to fluoroquinolones and to rifampicin. The remaining *P. aeruginosa* were considered as AS strains and were susceptible to third-generation cephalosporins and to carbapenems among ß-lactams, to tobramycin and amikacin among aminoglycosides, to ciprofloxacin and to fosfomycin. *P. aeruginosa* ATCC® 27853™, ATCC® 15692™, and ATCC® 53308™ were used as reference strains for the AS *P. aeruginosa* strains.

**Table 3:** Susceptibility of S. aureus and P. aeruginosa to antimicrobial agents by disc diffusion

Antibiotics																
	β-lactams		aminoglycosides		macrolides lincosamides		fluoroquinolones			tetracyclines	others					
*S. aureus* (number)	PEN	FOX	GEN	AMK	ERY	CLI	OFX	LVX	CIP	TGC	FAD	RIF	LZD	VNC		
MSSA (5)	R	S	S	S	S/I	S/I	S	S	S	S/R	S/R	S	S	S		
MRSA (5)	R	R	S/R	S/R	S/I/R	S/R	S/R	S/R	S/R	S	S/R	S/I/R	S	S		
	β-lactams									aminoglycosides			quinolones		others	
*P. aeruginosa* (number)	PIL	PTZ	TIC	TCC	CZD	FEP	ATM	IPM	MEM	AMK	GEN	TMN	CIP	NAL	RIF	FOF
AS (6)	S/R	S/R	S/R	S/R	S	S	I	S/I	S	S/I	S/R	S	S/I/R	R	R	S/R
MDRPA (5)	R	R	R	R	R	R	I/R	I/R	I/R	S/I/R	S/R	S/R	R	R	R	S/R

*AS* antibiotic susceptible, *MDRPA* multidrug-resistant *P. aeruginosa*, *MRSA* methicillin-resistant *S. aureus*, *MSSA* methicillin-susceptible *S. aureus, I* intermediate, *R* resistant, *S* susceptible, *AMK* amikacin, *ATM* Aztreonam, *CIP* ciprofloxacin, *CLI* clindamycin, *CZD* ceftazidime, *ERY* erythromycin, *FAD* fusidic acid, *FEP* cefepime, *FOF* fosfomycin, *FOX* cefoxitin, *GEN* gentamicin, *IMP* imipenem, *LVX* levofloxacin, *LZD* linezolid, *MEM* meropenem, *NAL* nalidixic acid, *OFX* ofloxacin, *PEN* penicillin, *PIL* piperacillin, *PTZ* piperacillin+tazobactam, *RIF* rifampicin, *TCC* ticarcillin+clavulanate, *TIC* ticarcillin, *TGC* tigecycline, *TMN* tobramycin, *VNC* vancomycin

### Antimicrobial assays of CAMPs

The MIC values of the peptides were determined by microbroth dilution [41, 42]. Bacteria were cultured in Mueller Hinton Broth 2 (MHB2, Cation-adjusted, Fluka Analytical) and grown overnight to exceed the turbidity of 0.5 McFarland. The obtained culture was diluted with sterile MHB2 to achieve a turbidity equivalent to 0.5 McFarland then diluted in MHB2 to give a final concentration of 2–7 × 10^5^ CFU/ml. Bacteria were incubated with different concentrations of the peptides; the highest tested concentration of each CAMP being 128 μg/ml. Since peptides have a tendency to bind polystyrene [42], mixtures were incubated at 37 °C in sterile 96-well polypropylene microtiter plates (Sigma-Aldrich) with shaking to prevent precipitation of CAMPs [43]. After 2 h of incubation, serial dilutions were plated onto Mueller Hinton Agar (MHA, MAST Group) plates and the number of colony-forming unit (CFU) was enumerated after incubation for 24 h at 37 °C. A control well without drugs was done for every strain. A reference strain was used in each test as a control to ensure reproducibility. After 16–24 h of incubation, MICs of CAMPs were determined. The MBCs were determined by plating out the contents of the first 3 wells showing no visible growth of bacteria onto MHA plates and were incubated for the following day. The MBC was defined as the lowest concentration of an antimicrobial agent that kills 99.9% of a particular organism [29, 42, 44].

### MICs of antibiotics and CAMPs combinations

The in vitro activities of the peptides (LL-37 and CAMA) in combination with colistin or imipenem were assessed against three clinical isolates of *P. aeruginosa*. The above obtained MICs of LL-37 and CAMA for each strain were used to calculate the concentrations needed for this experiment. Each strain was incubated with 1/10 or 1/5 its MIC of the peptide along with different concentrations of colistin or imipenem. After 16–24 h of incubation, the antibiotic MIC was determined for each combination experiment [30].

### Synergy studies by checkerboard technique

To highlight the enhanced activity of colistin and imipenem due to CAMPs combination, checkerboard assays were performed [45]. In brief, the experiments were done using 96-well microtiter plates containing CAMPs and antibiotics in two-fold serial concentrations. All plates were set up with increasing concentrations of CAMPs (LL-37 and CAMA) in the horizontal wells and antibiotics (colistin and imipenem) in the vertical wells. Bacterial suspensions were prepared to yield final inoculum of 2–7 × 10^5^ CFU/ml and added into the wells. The plates were incubated at 37 °C for 18–24 h and visually inspected for turbidity to determine the growth. The synergy interactions were evaluated by determining the FICI, calculated as follows:

$$ \mathrm{FICI}=\left(\mathrm{MIC}\ \mathrm{of}\ \mathrm{drug}\ {\mathrm{A}}_{\mathrm{combination}}/\mathrm{MIC}\ \mathrm{of}\ \mathrm{drug}\ {\mathrm{A}}_{\mathrm{alone}}\right)+\left(\mathrm{MIC}\ \mathrm{of}\ \mathrm{drug}\ {\mathrm{B}}_{\mathrm{combination}}/\mathrm{MIC}\ \mathrm{of}\ \mathrm{drug}\ {\mathrm{B}}_{\mathrm{alone}}\right). $$

FICI values were then interpreted as: synergy for FICI ≤0.5; additive for FICI between 0.5 and 1.5; indifference for values of FICI between 1.5 and 2; and antagonism was linked to values above 2 [46].

### LDH based cytotoxicity assay

Cell lysis due to treatment of IB3–1 and A549 cell lines by CAMPs alone or in combination with antibiotics was determined in vitro using the LDH based CytoTox 96®Non-Radioactive Cytotoxicity Assay according to manufacturer’s instructions (Promega). Cells were seeded in 12-well plates (TPP® tissue culture plates) in media and cultured for 1 day to obtain a concentration of 1.6× 10^6^ cells/ml (1 ml per well). Before treatment, supernatants were removed and 1 ml of serum-free medium was added to each well; cells were then rested for 1 h. To assess the cytotoxicity of CAMPs alone, cells were treated subsequently with two concentrations of LL-37 (32 and 64 μg/ml) and CAMA (8 and 16 μg/ml). To evaluate the cytotoxicity of the tested combinations, cells were treated with the highest concentrations of antibiotics and CAMPs for each combination. All treated cells were incubated for 24 and 48 h. After the desired incubation time, supernatants were aliquoted, centrifuged at 3500×g, 4 °C for 5 min to obtain cell-free samples and immediately analyzed for LDH. The dynamic range of the assay was determined using as control LDH release non-treated cells and as maximum LDH release cells lysed with Triton X-100. The average values of the culture medium background were subtracted from all values of experimental wells. The corrected values were used in the following formula recommended by the manufacturer to compute the cytotoxicity %.

$$ \mathrm{Cytotoxicity}\%={100}^{\ast}\mathrm{Experimental}\ \mathrm{LDH}\ \mathrm{release}\ \left(\mathrm{OD}490\right)/\mathrm{Maximum}\ \mathrm{LDH}\ \mathrm{release}\ \left(\mathrm{OD}490\right). $$

The treatment was considered not cytotoxic with less than 10% of LDH.

### Resistance induction in vitro

LL-37- and CAMA-induced resistance was assessed on two *P. aeruginosa.* The strains were grown independently in the presence of one half the MICs, diluted with MHB2, and inoculated for the next round of resistance induction for 28 days [47]. Resistance was assessed by determining the MICs of each antimicrobial agent on days 7, 14, 21, and 28 of treatment. The stability of induced-resistance was evaluated by determining the MICs after a single passage of each resistant strain in the absence of peptide. Gentamicin was used for comparison.

### Statistical analysis

MICs were determined in duplicate in 96-well microtiter plates. All experiments were performed in at least three independent assays and the data were analyzed with a general linear model procedure of *Statistical Package Software for Social Science* (SPSS, version 20.00, SPSS Institute Inc., Chicago, IL, USA). Statistically significant differences of MICs and MBCs between AS and MDR pathogens were further analyzed and compared using Chi-square and Fisher’s exact test. In colony count studies, results were presented as mean ± standard deviations between strains. Student and Mann-Whitney tests were used to compare the bactericidal activity of each concentration of the tested CAMPs on AS and MDR bacteria. Analyses of MIC variations due to different combinations were conducted using Wilcoxon and Paired t tests. Results of LDH based cytotoxicity assay were presented as the average of cytotoxicity % ± standard deviations between the three independent assays. Kruskal-Wallis test was used to evaluate the variation of cytotoxicity % compared to the control. *P* values ≤0.05 were considered statistically significant.

## Abbreviations

AS: Antibiotic susceptible
ATCC: American type culture collection
CAMA: Cecropin(1–7)-melittin A(2–9) amide
CAMPs: Cationic antimicrobial peptides
CFU: Colony-forming unit
Cytotoxicity %: Cytotoxicity percentage
DMEM: Dulbeccos modified Eagles medium
DNA: Deoxyribonucleic acid
ECCMID: European congress of clinical microbiology and infectious diseases
EUCAST: European committee on antimicrobial susceptibility testing
FBS: Fetal bovine serum
FICI: Fractional inhibitory concentration index
LDH: Lactate dehydrogenase
LL-37: Human cathelicidin
LPS: Lipopolysaccharide
MBCs: Minimal bactericidal concentrations
MDR: Multidrug-resistant
MDRPA: Multidrug-resistant *Pseudomonas aeruginosa*
MHA: Mueller hinton agar
MHB2: Mueller hinton broth 2
MICs: Minimal inhibitory concentrations
MRSA: Methicillin-resistant *Staphylococcus aureus*
MSSA: Methicillin-susceptible *Staphylococcus aureus*
OD: Optic density
*P. aeruginosa*: *Pseudomonas aeruginosa*
PBP: Penicillin binding protein
RNA: Ribonucleic acid
*S. aureus*: *Staphylococcus aureus*
SPSS: Statistical package software for social science
VDSA: Vancomycin-dependent *Staphylococcus aureus*
VRSA: Vancomycin-resistant *Staphylococcus aureus*
WHO: World health organization
